# Intravitreal Methotrexate

**DOI:** 10.18502/jovr.v16i4.9756

**Published:** 2021-10-25

**Authors:** Fatemeh Abdi, S. Saeed Mohammadi, Khalil Ghasemi Falavarjani

**Affiliations:** ^1^Eye Research Center, The Five Senses Institute, Rassoul Akram Hospital, Iran University of Medical Sciences, Tehran, Iran; ^2^Farabi Eye Hospital, Tehran University of Medical Sciences, Tehran, Iran; ^3^Stem Cell and Regenerative Medicine Research Center, Iran University of Medical Sciences, Tehran, Iran

**Keywords:** Inflammation, Intraocular Tumor, Intravitreal Injection, Methotrexate, Proliferative Vitreretinopathy, Uveitis

## Abstract

Intravitreal methotrexate (MTX) has been proven to be an effective treatment for various intraocular diseases. In this article, a comprehensive review was performed on intravitreal applications of methotrexate. Different aspects of the administration of intravitreal MTX for various clinical conditions such as intraocular tumors, proliferative vitreoretinopathy, diabetic retinopathy, age-related macular degeneration, and uveitis were reviewed and the adverse effects of intravitreal injection of MTX were discussed. The most common indications are intraocular lymphoma and uveitis. Other applications remain challenging and more studies are needed to establish the role of intravitreal MTX in the management of ocular diseases.

##  INTRODUCTION

Methotrexate (MTX), a Food and Drug Administration (FDA)-approved folic acid antagonist, inhibits DNA synthesis, repair, and subsequently cellular proliferation. MTX (formerly known as amethopterin) and its analog aminopterin were developed in 1947.^[1,2]^ Structural similarities of these drugs to folic acid and their ability to inhibit folate-dependent enzymes made them good choices for the treatment of cancers.^[3,4,5]^

MTX reduces the synthesis of polyamines by inhibition of dihydrofolate reductase (DHFR), an enzyme that catalyzes the reduction of dihydrofolate which results in decreased production of ammonia and hydrogen peroxide and lessens subsequent injury. MTX also increases the release of adenine nucleotides into the extracellular space which are converted to adenosine by cell-surface enzymes and exerts its inhibitory effects on nearly all inflammatory cells.^[5]^ Inhibiting the reduction of dihydrobiopterin to tetrahydrobiopterin by MTX results in the production of reactive oxygen species. Increased reactive oxygen species activate Jun N-terminal kinase which leads to activation of apoptosis and induction of cell cycle arrest.^[6]^ MTX modulates expression of lincRNA-p21, a long non-coding RNA, that regulates the p53-mediated apoptotic response without affecting the regulation of the cell cycle.^[4,7]^ Activation of signal transducer and activator of transcription proteins by receptor-associated Janus kinases (JAK-STAT signaling pathway) is also inhibited by MTX which leads to decrease in the production of inflammatory signals.^[8]^ MTX also has modulatory effects on the T-cells, monocytes, and fibroblast-like synoviocytes functions.^[9,10,11]^

MTX is one of the best choices in the treatment of systemic immune-mediated diseases such as rheumatoid arthritis (RA), psoriasis, juvenile idiopathic arthritis (JIA), multiple sclerosis (MS), systemic lupus erythematosus (SLE), and inflammatory bowel diseases (IBD). It is also useful for the prevention of graft rejection and treatment of malignant disorders due to its anti-inflammatory and immunomodulatory activities.^[2,12]^ Recently, promising results have been shown for the application of MTX in ophthalmic diseases. The effect of systemic MTX in the treatment of anterior, intermediate, posterior, and pan-uveitis, ocular mucous membrane pemphigoid, and scleritis has already been shown.^[13]^ However, intravitreal injection of MTX has been recently investigated widely as an approach to increase the drug availability to intraocular tissues and to decrease the systemic adverse effects. In this article, we reviewed the current evidence on the application of intravitreal MTX.

##  METHODS 

A PubMed and Scopus search was performed in October 2020 using each of the following keywords: “Methotrexate”, “MTX”, “Eye”, “Ocular”, “intravitreal MTX”, “intraocular MTX”, “intravitreal Methotrexate”, “intraocular Methotrexate” in different combinations. All article types including original articles, reviews, and case reports that described the ocular applications of MTX were identified. No limitation for the time of publication was applied. Abstracts only and non-English articles were excluded. All selected articles were reviewed thoroughly by the authors and relevant articles describing the application of intravitreal MTX were discussed.

##  RESULTS

Overall, 1066 articles were identified. From these, 146 articles described the use of intravitreal MTX. Intravitreal MTX was injected for intraocular tumors, uveitis, complex retinal detachment, diabetic retinopathy, age-related macular degeneration (AMD), and other indications.

### Preclinical Studies

Ericson et al^[14]^ investigated the safety profile of intravitreal MTX in rabbits. They injected 0.3 ml of 2.5–30 mg/ml concentration of MTX into the vitreous cavity of rabbits and showed that these concentrations would result in a flare in the anterior chamber, precipitation in the vitreous, and clouding of the lens. However, these concentrations are highly above routine intravitreal dose of MTX and side effects are much more likely with these concentrations. Ozkan et al^[15]^ evaluated the ultrastructural changes of retina induced by intravitreal MTX. Early changes were retinal edema, vacuolization, and disintegration of mitochondria of the retinal cells and long-term changes were edema in the photoreceptors and inner nuclear and ganglionic cell layers, three days and one month after four weekly injections of 800 µg intravitreal MTX. They showed that high dose intravitreal MTX results in significant ultrastructural changes in the rabbit retina in varying severities. In another study, Aly and Ebrahim^[16]^ assessed the effect of MTX toxicity on electroretinogram (ERG) and retinal caspase-3 activity which has an executive role in apoptosis of pigment epithelial cell, outer nuclear layer cell, and ganglion cells^[17]^ after injection of a single dose of 800 µg MTX. They showed that intravitreal injection of MTX leads to a significant reduction in a- and b-waves with an increase in caspase-3 activity. Hara and colleagues^[18]^ evaluated the effect of intravitreal MTX on embryonic stem cell differentiation and teratogenicity. They integrated embryonic stem cells into the retinas of adult mice and showed that injection of a single dose of intravitreal MTX four weeks after transplantation could increase neuronal differentiation, decrease expression of teratogenic markers, and reduce the proliferative activity of transplanted cells compared with non-treated retina. Palakurthi and colleagues^[19]^ evaluated the safety of MTX-loaded- biodegradable microneedle implants which were made using solving cast method. Implants were inserted into deep lamellar scleral pockets of both eyes of three rabbits. Animals were sacrificed and enucleated four weeks after implantation. Enucleated eyes were studied histopathologically for any evidence of inflammation or toxicity related to implant or embedded drug. No evidence of drug toxicity or inflammation and infection was seen around the implantation site and they showed that MTX-containing sustained release implant could be nontoxic and well tolerated by rabbit eyes.

Sunalp et al^[20]^ investigated the effect of intravitreal MTX on the experimental model of proliferative vitreoretinopathy (PVR). They showed that intravitreal injection of 250,000 homologous dermal fibroblasts into the rabbit vitreous with 10 nmol and 1 µmol concentration of MTX resulted in 71% and 83% retinal detachment, respectively, which was not lower than control cases. They proposed that as MTX inhibits DHFR, it is only effective on the fraction of cells that are at the S phase of the cell cycle and therefore active proliferation.^[21]^ This might explain the ineffectiveness of intravitreal injection of MTX in the reduction of PVR and subsequent retinal detachment.

Deng et al^[22]^ investigated the antimicrobial property of intravitreal MTX. They established a rabbit model of endophthalmitis induced by *Staphylococcus epidermidis* and assessed the effect of intravitreal injection of dexamethasone and MTX by grading the degree of vitreous haze and pathologic evaluation of ocular structures. The dexamethasone group had the most and the MTX group had the least intraocular inflammation and vitritis. Live bacteria were only isolated from the dexamethasone group and not in the MTX group. Pathologic evaluation revealed severe ocular destruction in the dexamethasone group and intact structures in the MTX group. They suggested that intravitreal MTX can reduce the risk of the development of bacterial endophthalmitis and associated ocular destruction compared with intravitreal dexamethasone.

Abbaszadeh Hasiri and colleagues^[23]^ evaluated the effect of intravitreal administration of MTX with two different doses for the treatment of endotoxin-induced uveitis (EIU) in the rabbit. They showed that mean histopathological inflammation intensity scores in both groups of intravitreal MTX (400 µg vs 800 µg) were significantly higher than the control group and intravitreal MTX did not have significant anti-inflammatory effects on EIU in rabbits.

### Clinical Studies 

MTX has been used to treat various ocular conditions. This part summarizes the clinical studies in which the intravitreal injection of MTX have been performed.

#### Intraocular tumors 

(i) Retinoblastoma

Retinoblastoma is the most common intraocular tumor in children.^[24]^ It was nearly 100% fatal about a century ago; however, therapeutic advances have led to a > 90% survival rate.^[25]^ Primarily enucleation was the method of choice for the treatment of retinoblastoma. Nowadays, this method is only reserved for unsalvageable eyes.^[26]^ The most prevalent drugs used for chemoreduction are vincristine, etoposide, and carboplatin followed by transpupillary thermotherapy, cryotherapy, and brachytherapy which cures > 90% of group A–C and about 50% of group D and E eyes with retinoblastoma.^[27]^ Kivela et al reported intravitreal MTX monotherapy for the management of intraocular relapse of retinoblastoma after chemoreduction in six eyes.^[28]^ They used an established protocol that was developed for primary intraocular lymphoma.^[29]^ Induction phase consisted of weekly intravitreal injections of 400 µg MTX for two months; consolidation and maintenance injections followed every two and four weeks for two and eight months, respectively.^[28]^ Objective response to intravitreal MTX alone occurred in five of six eyes.^[28]^ Their results are consistent with previous *in vitro* studies which showed that about one-third of retinoblastomas are sensitive to MTX.^[28,30]^

(ii) Intraocular lymphoma

Primary CNS lymphomas (PCNSL), characterized by an aggressive clinical course and poor outcome, are extranodal lymphomas arising exclusively inside the central nervous system and about 25% of these patients will develop vitreoretinal involvement.^[31]^ In the past three decades, the mean survival rate of PCNSL patients was significantly improved from 19.1% to 30.1% due to developments in chemotherapeutic agents.^[32]^ However, treatment protocols for intraocular lymphoma have not been fully validated and the need for low toxicity regimens that maintain high efficacy is undeniable.

Intraocular lymphomas are divided into four major groups, vitreoretinal lymphomas which are mostly high-grade B-cell lymphoma, primary choroidal lymphomas which are mainly low-grade B-cell lymphomas, secondary uveal lymphomas, and primary iridial lymphomas.^[33]^ Primary vitreoretinal lymphoma (PVRL), a great masquerader, is the most common and aggressive form of intraocular lymphoma and is usually associated with PCNSL. The characteristic feature of intraocular involvement of PCNSL is the presence of vitreous cells, especially in clumps which could be a masquerader of chronic nonresponsive uveitis.^[34]^ Several methods have been proposed for the treatment of intraocular involvement of PCNSL. Orbital radiation in combination with systemic chemotherapy with MTX was used and showed effectiveness; however, several studies reported ocular side effects such as cataract formation, dry eye and persistent corneal epithelial defects, radiation retinopathy, and optic neuropathy after orbital irradiation.^[35,36,37]^ In recent years, intravitreal MTX has been used more widely for the treatment of vitreoretinal involvement of PCNSL and has been found to be effective in induction of remission with acceptable morbidity.^[38,39,40]^ Fishburne and colleagues^[38]^ developed a protocol for the treatment of intraocular lymphoma by the intravitreal injection of 400 µg MTX. Intravitreal injection of 400 µg of MTX was performed twice weekly until the vitreous was clinically cleared of malignant cells, then weekly for one month, followed by monthly injections for one year. Seven eyes were treated based on this protocol which resulted in remission without any serious ocular adverse effects. Frenkel et al^[29]^ described their 10-year experience in treating vitreoretinal involvement of PCNSL by intravitreal injections of MTX. Their treatment protocol included injection of 400 µg MTX intravitreally twice weekly for four weeks, once weekly for eight weeks, and then once monthly for nine months. They reported their experience with 44 eyes of 26 patients. Sixteen patients were previously diagnosed with CNS or systemic lymphoma, and six were primarily diagnosed as chronic nonresponsive uveitis. Seventeen eyes completed the treatment protocol and 95% of the eyes were cleared from malignant cells and retinal infiltrates with a maximum of 13 injections (mean injections of 6.4). Those eyes which had poor initial visual acuity showed little improvement; however, those who had higher visual acuity showed more improvement in vision. Corneal epitheliopathy was the most common side effect that occurred in all patients. It appeared after the third injection and resolved when intervals between injections increased. Since there was no intraocular recurrence and no significant side effect, they proposed their protocol as a good first-line treatment option for intraocular lymphoma. Ma et al^[41]^ evaluated the outcomes of 19 patients with intraocular lymphoma who were treated with a combined intravenous high-dose MTX (6–8 g/m ${}^{2}$ ) and intravitreal MTX (400 µg). They reported that the patients with concurrent CNS involvement had worse therapeutic outcomes compared to those with isolated primary intraocular lymphoma who remained disease-free after salvage treatment. In another study, Smith and colleagues^[39]^ evaluated the effect of intravitreal MTX in the management of PCNSL involving the eye. All 26 eyes of 16 patients were cleared of malignant cells after a maximum of 12 MTX injections. Three patients experienced recurrence who were treated with another course of intravitreal MTX. The most common side effect in the study was the progression of preexisting cataracts, followed by corneal epitheliopathy, maculopathy, and vitreous hemorrhage.

(iii) Systemic lymphoma

Lymphoid proliferations can affect the intraocular structures in various ways. Involvement of ocular tissues by systemic lymphoma is rare and could masquerade benign ocular lesions.^[42]^ Lee Ong et al presented a patient with systemic chronic lymphocytic lymphoma and secondary anterior uveitis and hypopyon which was confirmed by anterior chamber tap and vitreous biopsy. As intravitreal triamcinolone in association with intrathecal MTX failed to improve the patient's vision, intravitreal MTX (400 µg) was injected weekly. Following the first injection, the patient's vision improved and the second injection resulted in the resolution of the hypopyon.^[43]^ Iris and ciliary body involvement are extremely rare in acute lymphoblastic leukemia (ALL). Mello and colleagues reported a patient with unilateral infiltration of iris and ciliary body by ALL which resulted in decreased vision and pseudohypopyon and iris irregularity. Anterior chamber reaction, iris, and ciliary body involvement was resolved after eight intravitreal MTX injections and the patient's visual acuity improved. Although the patient developed MTX-associated keratopathy, it was treated with frequent lubrication.^[44]^ Intraocular involvement of mycosis fungoides (cutaneous T-cell lymphoma) is an uncommon phenomenon. Reddy et al presented a case of mycosis fungoides with anterior chamber involvement, mutton fat keratic precipitate (KPs), and exudative retinal detachment which resulted in decreased vision. It was confirmed by vitreous biopsy and intravitreal MTX (400 µg) was administered weekly for one month and then every two weeks. This regimen resulted in the resolution of exudative retinal detachment and infiltrations. To prevent recurrence, the patient continued to receive monthly injection of intravitreal MTX.^[45]^ Ryan and coworkers reported a patient with systemic large B-cell lymphoma which presented with decreased vision and retinal pigment epithelial changes, as well as a yellow–white infiltrative macular lesion with adjacent retinal whitening and hemorrhage in the right and left eyes, respectively. Diagnostic vitrectomy confirmed the presence of large lymphoma cells with associated reactive lymphocytes. Although, PET scan, lumbar puncture, and bone marrow biopsy showed no evidence of malignancy, the patient received one treatment of intrathecal MTX (12 mg) at the time of the lumbar puncture in association with two treatment sessions of combination of intravitreal MTX (400 µg) and rituximab (1 mg) in her left eye at monthly intervals. Twenty-six months following initiation of the treatment, the patient showed no evidence of recurrence.^[46]^ Mantle cell lymphoma has poor long-term survival and is almost considered incurable.^[47]^ This aggressive non-Hodgkin lymphoma rarely involves the eye. Singer and colleagues reported a patient with mantle cell lymphoma who presented with decreased vision and bilateral optic disc swelling in the course of the disease. Resolution of optic disc swelling initiated in the temporal disk margin after intravitreal MTX injections with regression of the infiltrative process.^[48]^ In another study, Zhang et al reported a patient with metastatic large B-cell lymphoma which presented as recurrent iridocyclitis with mutton-fat KPs, hypopyon, and decreased vision in both eyes. Aqueous humor tap confirmed the diagnosis and the patient was planned to receive 25 intravitreal injections of 400 µg MTX which was administrated twice a week for four weeks; once a week for eight weeks; and then monthly for a total of nine months. In the course of treatment, patient received systemic high dose of MTX due to cutaneous relapse of lymphoma. Hypopyon was completely disappeared and only few cells were visible in the anterior vitreous cavity after the sixth injection. However, the patient experienced severe corneal toxicity; therefore, subsequent injections were cancelled. Epitheliopathy was treated with carboxymethyl cellulose sodium drops and patient's vision improved.^[49]^ Wickremasinghe and colleagues also reported two patients with systemic T-cell lymphoma which presented with fibrinous exudate in the anterior chamber and thickened and nodular iris. The first patient received one dose of intravitreal injection of MTX (400 µg) and responded well to therapy; five days after injection, the patient's vision improved, and uveitis and fibrinous exudate were resolved. The other case received three doses of intravitreal injection of MTX every six weeks and showed rapid improvement.^[50]^

#### Uveitis

Uveitis, intra-ocular inflammation of various causes, leads to irreversible visual loss if not treated properly.^[51]^ Treatments of infectious uveitis are mainly aimed at the pathogens; however, in noninfectious uveitis, corticosteroids are the main regimen to decrease inflammation.^[52]^ Local injections might be preferable in specific conditions, such as those patients without a concomitant systemic disease, those who are unable to take systemic therapy, or those who have very asymmetric ocular disease. Also, uveitic CME is one of the indications of local therapy. Local therapy leads to a high concentration of the drug at the site of disease activity and decreases the risk of systemic toxicity.^[53]^ Hardwig and colleagues^[54]^ evaluated the safety of intravitreal MTX for improvement of ocular diseases such as uveitis and advanced proliferative diabetic retinopathy (PDR), epithelial downgrowth, and idiopathic fibrovascular proliferation. Patients were treated with a single dose of 400 µg intravitreal MTX. Seven of nine uveitis eyes showed improvement in visual acuity, one remained stable, and one patient had decreased visual acuity which seemed to be due to the advanced preexisting pathology and natural history of the disease rather than the treatment intervention. In another retrospective case series, Taylor and colleagues^[55]^ showed that a single injection of 400 µg intravitreal MTX in patients with unilateral noninfectious intermediate, posterior, or panuveitis and/or CME could lead to improvement of ocular inflammation, visual acuity, CME, and reduction of systemic immunosuppressive therapy. Five of the fifteen patients experienced uveitic flare after a median of four months. Four patients who achieved a partial improvement of CME after injection of intravitreal MTX showed no further improvement following IVTA injection. In a larger multicenter interventional case series, Taylor et al^[56]^ confirmed the result of the previous study. They showed that intravitreal injection of 400 µg MTX could effectively improve visual acuity and/or reduce CME in 30 of 38 eyes. In some patients, it also reduced the need for systemic immunosuppressive drugs. In another study, Khalil and coworkers,^[57]^ evaluated the efficacy of intravitreal MTX in controlling posterior segment involvement of Behcet's disease (BD) in comparison to retrobulbar triamcinolone acetonide (TA). They showed that improvement of anterior chamber reaction and vitreous inflammation was similar between the two groups; however, relapses were noted less in patients who received intravitreal injection of 400 µg MTX. They suggested that intravitreal MTX has a promising result and may ensure better control of the inflammatory reaction and longer remission in comparison to retrobulbar TA in BD patients. In a similar study by Bae et al,^[58]^ the effect of intravitreal MTX in the treatment of refractory retinal vasculitis due to BD was evaluated. Intravitreal injection of 400 µg MTX was given monthly until visual acuity and intraocular inflammation were stable. Patients experienced significant improvement in visual acuity, decrease in fluorescein leakage, and levels of aqueous humor's interleukin (IL)-6 and IL-8, four weeks after intravitreal MTX without any significant change in IOP. As the increase in levels of IL-6 and IL-8 is associated with refractory retinal vasculitis in BD, intravitreal MTX could be effective in these patients.

Serpiginous choroiditis is defined as chronic, progressive, and recurrent inflammation of the choroid that leads to loss of choriocapillaris and atrophy of RPE and photoreceptors.^[59]^ In some patients, there is an association with underlying mycobacterium tuberculosis infection and hypersensitivity to its components.^[60]^ This condition usually responds well to antitubercular therapy and systemic corticosteroids; however, inflammatory damage may limit visual outcomes and continuation of antibiotics with the addition of immunomodulatory drugs such as MTX may result in disease quiescence.^[61]^ Tsui et al^[62]^ reported a patient with serpiginous-like choroiditis (SC), from a TB-endemic area and positive QuantiFERON assay which was refractory to anti-tuberculosis regimen and systemic corticosteroids. Within six weeks of starting anti-TB medication, the patient started receiving azathioprine 150 mg daily. Despite receiving antibiotics, prednisolone, and azathioprine, steady SC extension was seen. Therefore, intravitreal MTX was injected and subcutaneous MTX was prescribed. Following the second intravitreal injection of MTX, the progression of the lesion stopped and borders of the lesion started to contract. The lesion remained quiescent 24 months after treatment. In another study, Sahin et al^[63]^ investigated the antiproliferative and anti-inflammatory effect of intravitreal MTX on suppressing intraocular inflammation in two patients with presumed tuberculosis-related uveitis. Both patients received 400 mg of intravitreal MTX at the eighth week of anti-tuberculous therapy and showed improvement of visual acuity, suppression of intraocular inflammation, and resolution of CME. No recurrence was observed eight months after cessation of the anti-tuberculous regimen. Chin et al^[64]^ reported a patient who had latent extrapulmonary tuberculosis with choroidal granulomas (tuberculomas) that had been treated with antitubercular therapy one year earlier. Systemic prednisolone provided visual improvement; however, avascular necrosis of the hip complicated the therapy. As cessation of systemic corticosteroid led to worsening of visual symptoms in this patient, diagnostic pars plana vitrectomy (PPV) in addition to intravitreal injection of MTX (400 µg) was performed. Visual acuity improved after surgery and an intravitreal dexamethasone implant was inserted one week later which resulted in sustained resolution of choroidal infiltration.

Viral retinitis is a rare ocular infection that usually results in high rates of visual impairment. While acute retinal necrosis (ARN) occurs in immunocompetent patients, progressive outer retinal necrosis (PORN) and cytomegalovirus (CMV) retinitis usually happen in immunocompromised patients.^[65]^ The human herpesvirus family includes herpes simplex virus, varicella-zoster virus (VZV), Epstein-Barr virus (EBV), and CMV. CMV infection is the leading viral cause of visual impairment in immunocompromised patients, especially those who had undergone organ transplants. Huang et al^[66]^ reported a patient who had acute myeloid leukemia (AML) and developed bilateral CMV retinitis with bilateral CME, and optic disc swelling in the right eye six months following bone marrow transplantation. Four weekly injections of intravitreal MTX (400 µg) were performed in the right eye which was more severely affected in combination with oral valganciclovir. Visual acuity improved, optic disc swelling was resolved, and macular edema was subsided in the MTX-injected eye one month after injection. However, the non-injected eye showed no sign of resolution of macular edema. No recurrence of macular edema was observed in the right eye during the next eight months of follow-up. Mashima and colleagues^[67]^ presented a patient with interstitial pneumonia and chronic pyelonephritis who had been on methylprednisolone for 20 years. The patient developed vitreous opacity and extensive necrotizing retinitis with retinal hemorrhage sparing the posterior pole. Polymerase chain reaction (PCR) of the vitreous sample was positive for EBV but negative for HSV, VZV, and CMV. The patient was unresponsive to intravenous ganciclovir and intravenous acyclovir, therefore, intravitreal MTX (400 µg) was tried for the patient. Three days after intravitreal injection of MTX, the white–yellowish lesion in the ocular fundus was regressed. In line with regression of the lesion, copy numbers of EBV-DNA in aqueous humor were also decreased.

#### Proliferative vitreoretinopathy (PVR)

PVR is defined as the growth of fibroglial tissue on both sides of the detached retina and posterior hyaloid face.^[68]^ It may be presented as low as the presence of cellular debris in the vitreous cavity to the presence of full-thickness retinal folds.^[69]^ PVR occurs in 5–10% of cases of rhegmatogenous retinal detachments (RRD) and is the main cause of surgical failure after the repair of RRD.^[70]^ Several studies have shown the role of inflammation in the pathogenesis of PVR and reported the effectiveness of corticosteroids on inhibiting the development of PVR.^[71,72]^ Denstedt and colleagues,^[73]^ reported a case of a needle penetrating injury of the globe which resulted in retinal detachment and progressive post-traumatic PVR. The patient underwent multiple vitrectomies in association with multiple intravitreal injections of 200 μg MTX every two to three weeks. The patient's best-corrected visual acuity (BCVA) was 20/40 and the retinal periphery was flat with stable fibrosis in the last follow-up, 15 months after the initial treatment. Benner et al^[74]^ treated five eyes with severe PVR and recurrent retinal detachment using relaxing retinectomy, extended perfluorocarbon liquid tamponade for four weeks, and a series of intravitreal MTX injections (100–200 μg) every two weeks. Injections started within one week after re-detachment surgery. Patients were followed for 11–27 months and interestingly, all patients remained attached and four eyes recovered to ambulatory vision ( > 20/200) with normal intraocular pressure (IOP). This study showed that multiple intravitreal MTX injections could be beneficial for treating complex retinal detachment associated with PVR. Ghasemi Falavarjani et al^[75]^ evaluated the role of intra-silicone oil (SO) injection of MTX at the end of vitrectomy surgery for RRD with associated PVR. They injected 250 µg MTX into the silicone oil at the end of the surgery in 22 eyes of 22 patients with RRD associated with grade C PVR and compared the rate of retinal detachment with 22 eyes of 22 control patients. They showed that the rate of re-detachment was lower among the MTX group, although the difference was not statistically significant. Nourinia and coworkers^[76]^ evaluated the effect of repeated intra-silicone oil injections of MTX on the outcomes of surgery for RRD associated with grade C PVR. At the end of the vitrectomy and intraocular injection of SO, and at the 3 ${}^{\mathrm{rd}}$ and 6 ${}^{\mathrm{th}}$ week after the surgery, the patient received 250 μg of intravitreal MTX. Eleven eyes were treated and followed for about nine months; the retina of all treated eyes remained attached and BCVA was significantly improved at the last follow-up visit. They stated that repeated intra-silicone injection of MTX could be a promising adjunctive procedure for the treatment of RRDs complicated by PVR. Sadaka et al^[77]^ evaluated the effect of intravitreal MTX infusion during PPV for retinal detachment in patients with high risk for the development of PVR (severe recurrent PVR with tractional retinal detachment [TRD] and/or a history of severe ocular inflammation). A mixture of 500 mL balanced saline solution with added 40 mg of MTX was used as an infusion bottle during the surgery of 29 eligible patients. They believed that this solution yields intraocular MTX levels equivalent to that used in intraocular lymphoma (400 μg intravitreal injection).^[54,78]^ Six months after the treatment, 83% of the patients had stable or improved BCVA and 90% of the retinas remained attached. Therefore, they suggested that intravitreal infusion of MTX during the surgery could be beneficial for those eyes which are at high risk for PVR development due to a history of prior PVR or intraocular inflammation. There is also an ongoing phase 3 clinical trial regarding the effectiveness of intravitreal MTX on the rate of re-detachment due to PVR that requires surgery. During Gain Understanding Against Retinal Detachment (GUARD) trial, patients with recurrent retinal detachment due to PVR with star folds in at least three cumulative clock hours documented on retinal imaging, or for retinal detachment associated with open globe injury are enrolled. At the end of the vitrectomy or on first postoperative day, and then weekly for eight weeks, followed by every-other-week treatment through the 16 ${}^{\mathrm{th}}$ postoperative week, patients receive ADX-2191 (intravitreal MTX 0.8%, Aldeyra therapeutics) injections.^[79]^

#### Proliferative diabetic retinopathy 

Ghasemi Falavarjani et al^[80]^ evaluated the effect of intra-silicone oil injection of MTX at the end of vitrectomy for advanced PDR and assessed the rate of retinal re-detachment associated with fibrovascular proliferation or PVR. They injected 250 µg MTX intravitreally into 19 eyes with severe diabetic tractional macular detachment or combined tractional or rhegmatogenous retinal detachment and compared the outcomes with 19 eyes of a control group. Retinal re-detachment with fibrovascular proliferation or PVR occurred in seven eyes (36.8%) in the MTX group and eight eyes in the control group which was not statistically significant. Therefore, they showed that intra-silicone injection of MTX at the end of vitrectomy for retinal detachment associated with severe PDR did not reduce the risk of postoperative retinal detachment due to fibrous or fibrovascular proliferation. In another study, Hardwig et al^[54]^ reported five patients with PDR associated with TRD or CME. Three patients received 200 µg and two were treated with a total of 400 µg of intravitreal MTX. Two patients with PDR and TRD experienced decreased vision, two patients with PDR and TRD/DME showed increase in vision, and one patient with PDR and TRD experienced no change in visual acuity.

#### Diabetic macular edema (DME)

Inflammation plays an important role in the pathogenesis of DME. Intraocular inflammatory mediators increase in the course of the disease.^[81]^ Several studies have shown that intravitreal injection of corticosteroids results in improvement of DME mainly through suppressing the inflammatory mediators.^[81,82,83]^ However, glaucoma and cataract formation are major drawbacks in intravitreal corticosteroid injections.^[83]^ Intravitreal MTX has been investigated as an anti-inflammatory agent in the treatment of persistent DME. In a prospective interventional case series, intravitreal MTX (400 µg) was injected in 18 eyes of 16 patients with persistent center-involving DME unresponsive to at least three consecutive intravitreal bevacizumab (IVB) injections or two consecutive bevacizumab injections plus macular photocoagulation. Intravitreal injection of MTX resulted in statistically significant anatomical and visual improvement.^[84]^

In another study, the efficacy of IVB combined with intravitreal MTX (IVM) in the treatment of DME was investigated. Thirty-six eyes of 18 patients were randomly allocated into the two groups to receive three monthly injections of IVB (1.25 mg) plus IVM (400 µg) or IVB alone. In contrast to the previous study, no significant therapeutic effects for IVB combined with IVM compared to IVB alone were seen over a three-month follow-up.^[85]^

#### Age-related macular degeneration

Although vascular endothelial growth factor (VEGF) is the main mediator in neovascular AMD, other inflammatory mediators play a significant role in its pathogenesis. An increase in reactive oxygen species which can cause cellular damage, caspase activation inducing cell death, complement activation, breakdown of Bruch's membrane and retinal pigment epithelium (RPE) by matrix metalloproteinases, and production of cytokines are the major inflammatory reasons for neovascular activity in AMD.^[86]^ Therefore, interrupting the angiogenesis cascade by inhibiting inflammatory agents may prove effective in the treatment of neovascular AMD. Kurup et al^[87]^ treated two patients who were refractive to conventional anti-VEGF therapy. They injected 400 µg intravitreal MTX and at the two-week follow-up visit, visual acuity improved, and perifoveal subretinal fluid and leakage were decreased. As safety indices are unknown, they proposed that this treatment should be used for selected patients who are refractory to traditional treatments. Soheilian and coworkers^[88]^ assessed the effect of combined intravitreal MTX and bevacizumab on choroidal neovascularization in AMD. They injected 400 µg of MTX combined with 1.25 mg of bevacizumab. The mean visual acuity improved and the mean central macular thickness (CMT) decreased. They suggested that the addition of intravitreal MTX to bevacizumab is safe and may enhance the therapeutic effect of bevacizumab for regression of neovascular complex. They proposed that MTX may also decrease the development of fibrous component and scar formation based on OCT and fundus images.

#### Epithelial downgrowth

Epithelial downgrowth, characterized by intraocular migration of epithelial cells, can lead to endothelial decompensation, angle-closure glaucoma, intractable pain, and TRD.^[89]^ Several methods such as membrane peeling, argon laser, excision of affected intraocular structures, and fluorouracil injection have been reported for treatment of this condition;^[90]^ however, more than half of these patients are unresponsive to treatment.^[89]^ Lambert et al^[91]^ reported the use of multiple intravitreal injection of MTX for treatment of a patient with recurrent epithelial downgrowth. The patient was followed for 14 months after his last injection and neither recurrence nor any side effect was seen. In another study, a patient who was suffering from epithelial downgrowth due to previous radial keratotomy and trabeculectomy was treated successfully with the injection of 400 µg intravitreal MTX every two weeks for six doses. Epithelial downgrowth was successfully resolved and no remnant of the epithelial downgrowth was visible.^[92]^ Hardwig reported a patient with idiopathic fibrovascular proliferation for whom intravitreal injection of 200 µg MTX was done. The patient experienced four lines increase in visual acuity three months after the treatment.^[54]^

### MTX Toxicity

Corneal epitheliopathy as the most common adverse effect of intravitreal injection of MTX is believed to be due to the local spillage of MTX into the subconjunctival space resulting in damage to the limbal stem cells which causes a transient limbal stem cell deficiency, and therefore corneal haze and epithelial breakdown.^[93]^ In some studies, all patients develop some form of keratopathy, ranging from diffuse punctate keratopathy to severe epitheliopathy, which usually appears after the third injection. These patients usually respond well to short courses of topical lubricants, topical steroids, and increasing the interval between injections. Topical folinic acid 0.003% and systemic folic acid 1 mg once daily could be effective in those who were unresponsive to lubrication.^[29,39,93]^ Zhou et al attempted to reduce the incidence of keratopathy caused by intravitreal MTX. They divided patients into two groups. Group A received intravitreal MTX at a dosage of 400 μg twice a week for the first four weeks, weekly for the following eight weeks, and then monthly for the last nine months. Patients in group B were started on the treatment protocol described above and switched directly to monthly injection for nine months when ocular remission was achieved. They showed that with a reduced injection frequency, the incidence of keratopathy could be lowered by about 60% without ocular recurrence during follow-up.^[94]^ In another study, Sahay et al reported a patient with diffuse large B-cell lymphoma and ocular involvement which was treated with weekly injections of intravitreal MTX in both eyes. The patient developed severe photophobia, tearing, and a decrease in vision due to severe limbitis with annular corneal epitheliopathy and corneal haze. Further injections were discontinued and the patient received topical lubricants, cyclosporine, loteprednol, folinic acid, and systemic folic acid which resulted in complete resolution at two-week follow-up.^[95]^ Ghasemi Falavarjani and colleagues evaluated the effect of intravitreal MTX injection (400 µg) on corneal endothelial cells in eyes with persistent DME. They assessed corneal endothelial cell features using specular microscopy. After six months of follow-up, they showed that intravitreal injection of 400 µg MTX had no significant effect on corneal endothelial cell measurements.^[96]^ They did not observe clinically significant keratopathy in their series. Progression of preexisting cataract is also noted as a side effect of intravitreal MTX injection, however, the majority of evaluated patients were vitrectomized that predisposed the eyes to the progression of cataract.^[39]^ Band keratopathy and iris and anterior chamber angle neovascularization with subsequent neovascular glaucoma were also reported as a rare complications of intravitreal injection of MTX.^[29]^ Choudhury et al reported two patients, one having idiopathic retinal vasculitis and the other having pars planitis in association with CME who received intravitreal injection of MTX. Following intravitreal injections, both patients experienced pain and a decrease in vision within 24 hr of receiving intravitreal MTX injection. Vitreous samples and cultures of used and unused vials of MTX from the same batch grew *Ralstonia pickettii*. Patients received intravitreal injection of vancomycin, amikacin, and dexamethasone with the diagnosis of acute endophthalmitis which resulted in visual recovery. This outbreak was caused by drug contamination at the compounding pharmacy.^[97]^ Hardwig and colleagues showed that intravitreal MTX is safe for non-PCNSL indications, as there was only one case among 16 treated patients who developed corticosteroid-responsive sterile endophthalmitis.^[54]^ In another study, Hardwig et al evaluated the visual results of intra-silicone injection of MTX at the time of or after the surgery for retinal detachment. They suggested that cumulative dosages of MTX ranging from 200 µg to 1200 µg is safe as there were no adverse effects observed following single injection or serial injections.^[98]^ Maculopathy, vitreous hemorrhage, and optic atrophy were also reported as rare complications of intravitreal MTX.^[39]^

##  DISCUSSION

Intravitreal MTX has been used in several ophthalmic conditions such as intraocular tumors, DME, AMD, PVR, uveitis, and epithelial downgrowth, showing promising results in many of these conditions. While the treatment is highly effective in intraocular lymphoma and uveitis, the efficacy in other diseases remains to be confirmed.

MTX, a steroid-sparing agent, is becoming more popular and even the first-choice drug in some conditions that require long-term immunosuppression.^[99]^ MTX exerts its anti-inflammatory and immunomodulatory effects through several mechanisms such as inhibition of DHFR, increasing level of intracellular and extracellular adenosine, inhibiting the reduction of dihydrobiopterin to tetrahydrobiopterin, modulation of expression of lincRNA- p21, inhibition of JAK-STAT signaling pathway, and modulation of functions of T-cells and monocytes.^[2]^ The mechanism of action of the intravitreal MTX is believed to be similar to its administration for other systemic diseases.^[2,100]^

Intravitreal MTX is considered safe with the current doses. The most common side effect is corneal epitheliopathy after repetitive injections which is easily treated with frequent lubrication and increasing the interval between injections. Few other side effects such as progression of preexisting cataracts, neovascular glaucoma, maculopathy, vitreous hemorrhage, and corneal endotheliopathy are very rare and the Causal associations are unclear.

Intravitreal MTX is generally well tolerated and avoids the side effects of systemic administration of MTX or alternative drugs. Although current routine clinical applications are limited to some forms of intraocular tumors and uveitis, the use of intravitreal MTX needs further investigations for other clinical applications. Different studies have shown some promise for intravitreal MTX in diabetic retinopathy, AMD, and after retinal detachment surgery; however, larger, randomized, multi-center clinical trials with long-term follow-ups are required to clarify the benefits of intravitreal MTX in different vitreoretinal diseases.

##  Financial Support and Sponsorship

Nil.

##  Conflicts of Interest

The authors do not have any conflicts of interest.
